# TVNet: Multimodal medical image fusion by dual-branch network with vision transformer and one-shot aggregation

**DOI:** 10.1177/00368504251375188

**Published:** 2025-11-04

**Authors:** Jianguo Wang, Wenran Jia, Yuhang Liu, Pengfei Wu, Peng Geng, Xuguang Meng

**Affiliations:** 1 School of Industrial Internet, Beijing Information Technology College, Beijing, China; 2 School of Information Science and Technology, Shijiazhuang Tiedao University, Shijiazhuang, China; 3 Department of Radiology and Diagnostic Imaging, 3158 University of Alberta, Edmonton, Alberta, Canada; 4 Department of Technology Development, Hebei Information Industry Technology Co., Ltd., Shijiazhuang, China

**Keywords:** Medical image fusion, convolution neural network, vision transformer, long-range dependencies, multiscale features

## Abstract

The task of medical image fusion involves synthesizing complementary information from different modal medical images, which is of very significant for clinical diagnosis. The existing medical image fusion algorithms overly rely on convolution operations and cannot establish long-range dependencies on the source images. This can lead to edge blurring and loss of details in the fused images. Because the Transformer can effectively model long-range dependencies through self-attention, a novel and effective dual-branch feature enhancement network called TVNet is proposed to fuse multimodal medical images. This network combines Vision Transformer and Convolutional Neural Network to extract global context information and local information to preserve detailed textures and highlight the structural characteristics in source images. Furthermore, to extract the multiscale information of images, an enhancement module is used to obtain multiscale characterization information, and the two branches information are efficiently aggregated at the same time. In addition, a hybrid loss function is designed to optimize the fusion results at three levels of structure, feature, and gradient. Experiment results prove that the performance of the proposed fusion network outperforms seven state-of-the-art methods in both subjective visual effects and objective metrics. Our code is available at https://github.com/sineagles/TVNet.

## Introduction

Different modal images contain different information that plays different roles in clinical diagnosis. For example, computed tomography (CT) images provide hard tissue imaging with large density differences. But soft tissue information is poor. Magnetic resonance imaging (MRI) contains high-resolution details of soft tissue anatomy but lack information about bone tissue. In contrast, single-photon emission CT (SPECT) and positron emission tomography (PET)^1^ offer insights into functional aspects such as blood flow and metabolic changes, but they have limited spatial resolution. The single modal medical image is difficult to provide sufficient information for the clinical diagnosis. Inspired by techniques such as dynamic weight optimization^2^ and attention enhancement fusion,^3^ the fusion of typical and complementary information from CT, PET, MRI, and SPECT into one image can more comprehensively reveal pathological features and enrich the representation of tissue physiology and pathological status, thereby helping doctors make accurate diagnoses.^4^

Traditional medical image fusion methods are typically divided into spatial domain, multiscale, and hybrid approaches. Spatial domain methods, such as principal component analysis^5^ and differential evolution.^6^ However, this method directly weights the pixel values of the source image, which often leads to edge blurring. Multiscale transforms, such as the pyramid transform,^7^ dual-tree complex wavelet transform,^8^ and contourlet transform,^9–11^ can enhance edge and texture preservation. However, they require careful selection of decomposition layers. To improve fusion performance, hybrid approaches have been explored, including combining multiscale transforms with sparse representation,^12^ and combining spiking cortical models with total variational decomposition.^13^ Although these approaches are effective, they are generally complex and have limited scalability.

Deep learning–based image fusion models exhibit strong feature extraction capabilities. Convolutional Neural Networks (CNNs) are widely used to extract local image features and generate fused outputs. DeepFuse^14^ introduced a CNN for multiexposure fusion, but its simple structure limits feature utilization. To improve this, Li et al.^15^ added dense connections, although only single-scale features were considered. Song et al.^16^ addressed this with multiscale filters in DenseNet. PMGI^17^ reframed fusion as a proportional preservation task but suffered from artifacts. Zhang et al.^18^ proposed an adaptive decision module to generate sharper images, Huang et al.^19^ used attention mechanisms for disentangled representation but faced brightness issues.

Although deep learning–based methods generally outperform traditional techniques, they still face challenges: (1) CNNs have limited receptive fields, restricting long-range feature extraction; (2) most methods rely on single-scale features, which are inconsistent with the human visual system; (3) poorly designed loss functions often lead to blurred structures and degraded fusion quality. To address these issues, we propose a dual-branch fusion model that integrates CNN and Transformer architectures. The main contributions are as follows:

1. We design a dual-branch feature extractor that integrates a Vision Transformer (ViT) with a CNN enhanced by One-Shot Aggregation (OSA). The OSA module efficiently aggregates multilayer features to retain fine details, while the Transformer captures long-range dependencies.
2. The proposed hybrid loss function combines pixel-level, structural, and gradient information to guide the network in preserving lesions and texture details.
3. To capture multiscale features in line with human visual perception, we introduce a Sub-pixel Context Enhancement (SCE) module that effectively fuses local and global information.
4. The proposed TVNet achieves a better fusion effect in extensive experiments with various medical image pairs.

## The proposed TVNet

**Figure 1.**
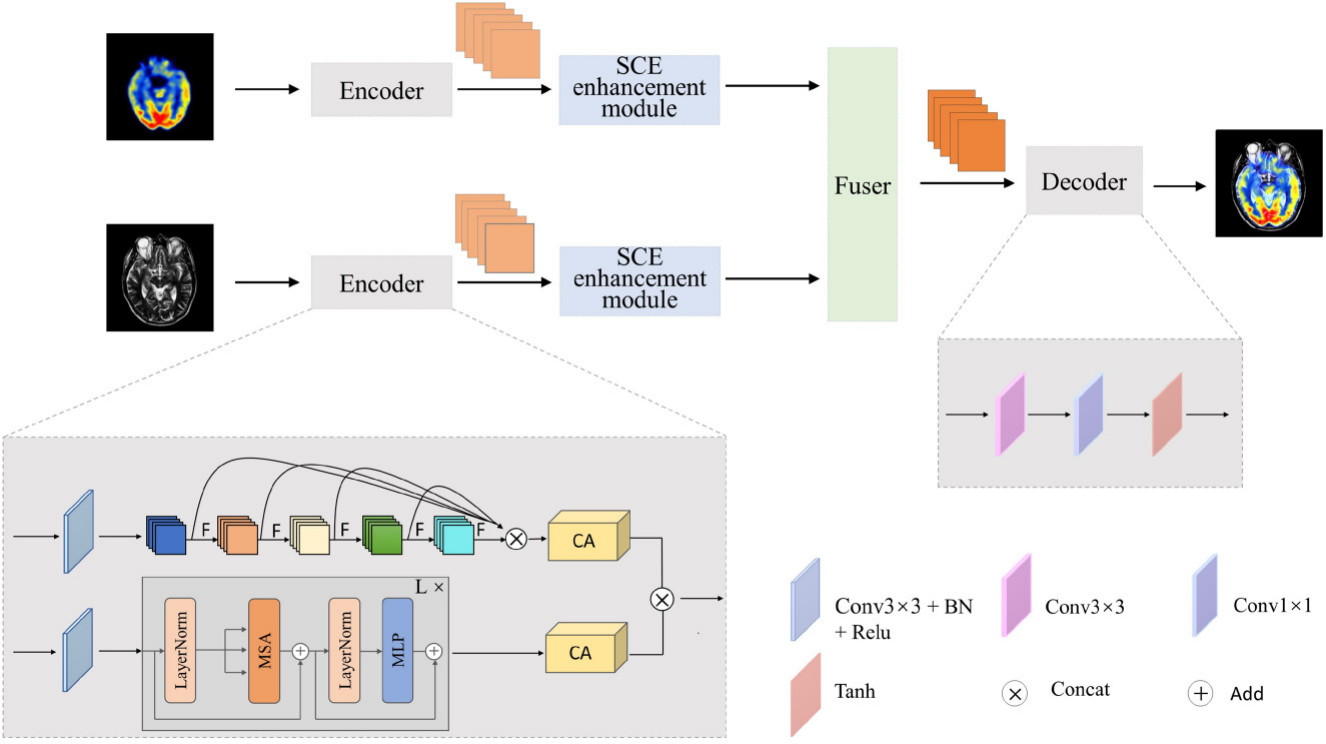
Architecture of the proposed TVNet.

The proposed TVNet is shown in Figure 1. Like most of medical image fusion networks, the proposed model includes the encoder, the fusion layer, and the decoder. In the proposed TVNet, we designed a dual-branch encoder, where the CNN branch is constructed using the OSA module. Unlike traditional CNNs, which either perform shallow stacking or dense connections prone to feature redundancy, the OSA block aggregates multilayer convolutional outputs in a one-shot manner. This design enhances local semantic integration, effectively retaining critical structural features such as edges, textures, and anatomical contours, which are essential in medical image fusion. Moreover, the ViT branch is introduced in parallel to capture global contextual dependencies, enabling the model to better align semantic information across different modalities. The proposed encoder combines OSA, and Transformer provides a complementary fusion mechanism. It is worth noting that both source images are processed through the same encoder framework, with each image being fed separately into its own CNN branch and Transformer branch for feature extraction.

Subsequently, the Coordinate Attention (CA) mechanism is introduced to enhance feature representation of the CNN branch and the Transformer branch. Unlike conventional methods that treat all feature channels equally, the CA mechanism lays in its direction-aware and position-sensitive encoding, which efficiently captures cross-channel relationships and long-range spatial dependencies, significantly improving the semantic expressiveness of features. After optimization with the CA mechanism, the feature maps are further fused through element-wise multiplication to achieve deep integration of information across branches. The fused features are further fed into the SCE module. This module employs three parallel branches to extract and integrate multiscale features, effectively capturing semantic information at various scales and aligning with the multiresolution perception characteristics of the human visual system.

In fusion strategy, we adopt the addition strategy to fuse the features extracted from two encoder branches. To reconstruct the final fused image, we design a lightweight decoder using a combination of convolution layers and activation functions, enabling effective dimensionality reduction while preserving fine structural details.

### Encoder

The extracted features of the encoder in the fused network will significantly affect the visual effect of final fused images. Therefore, to adequately extract texture detail features, global features and multiscale features of the source images, OSA module, Transformer module, attention mechanism, and enhancement module are used in the encoder to obtain richer feature information.

#### One-shot aggregation module

**Figure 2.**
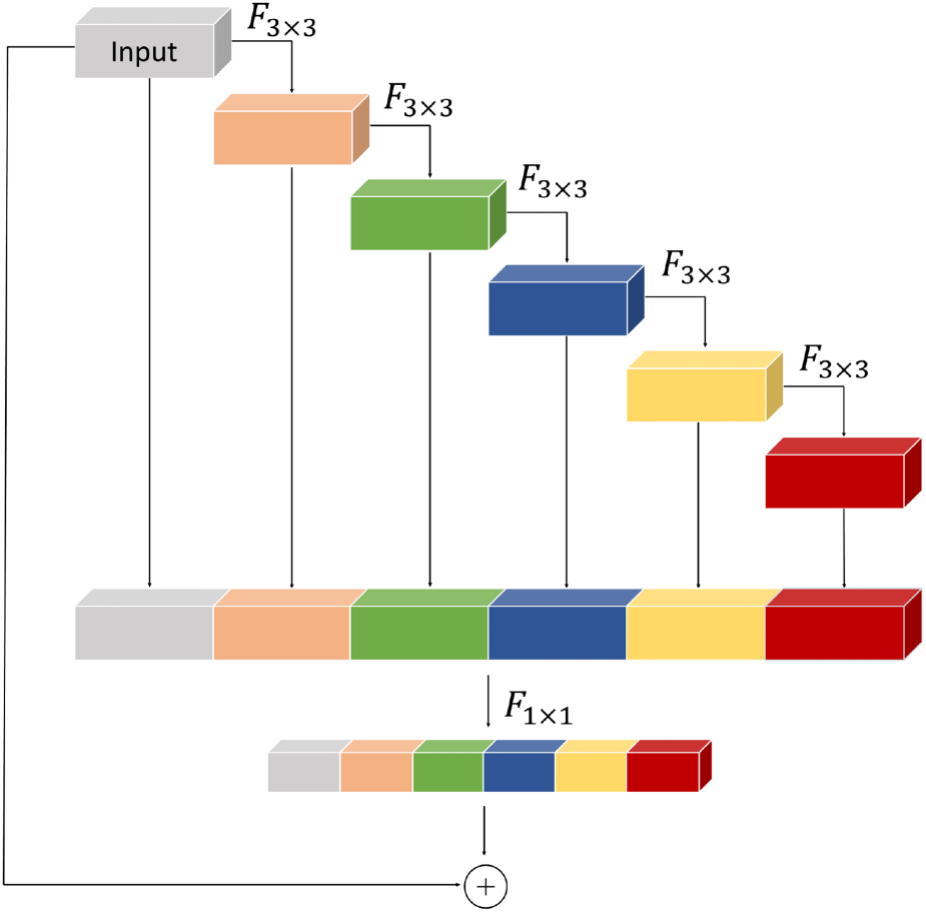
One-Shot Aggregation (OSA) module.

To help the encoder capture fine-grained texture details more effectively, the OSA block from VoVNet^20^ is introduced to construct the CNN branch in the proposed method. The architecture of OSA module is depicted in Figure 2. The OSA module aggregates the extracted features from different layers, making the aggregation in the final layer more intense and enriching the information of source images. To be specific, we first perform five convolution operations using a 3 × 3 convolution kernel on the input source images. In this process, the output after the last convolution is used as the input of the next convolution. Then, the result of the final layer is concatenated with the feature maps of all previous layers. Finally, the channel count of the input feature maps is adjusted by 1 × 1 convolution, and the skip connection helps recover information that may be lost during feature extraction.

#### Transformer module

**Figure 3.**
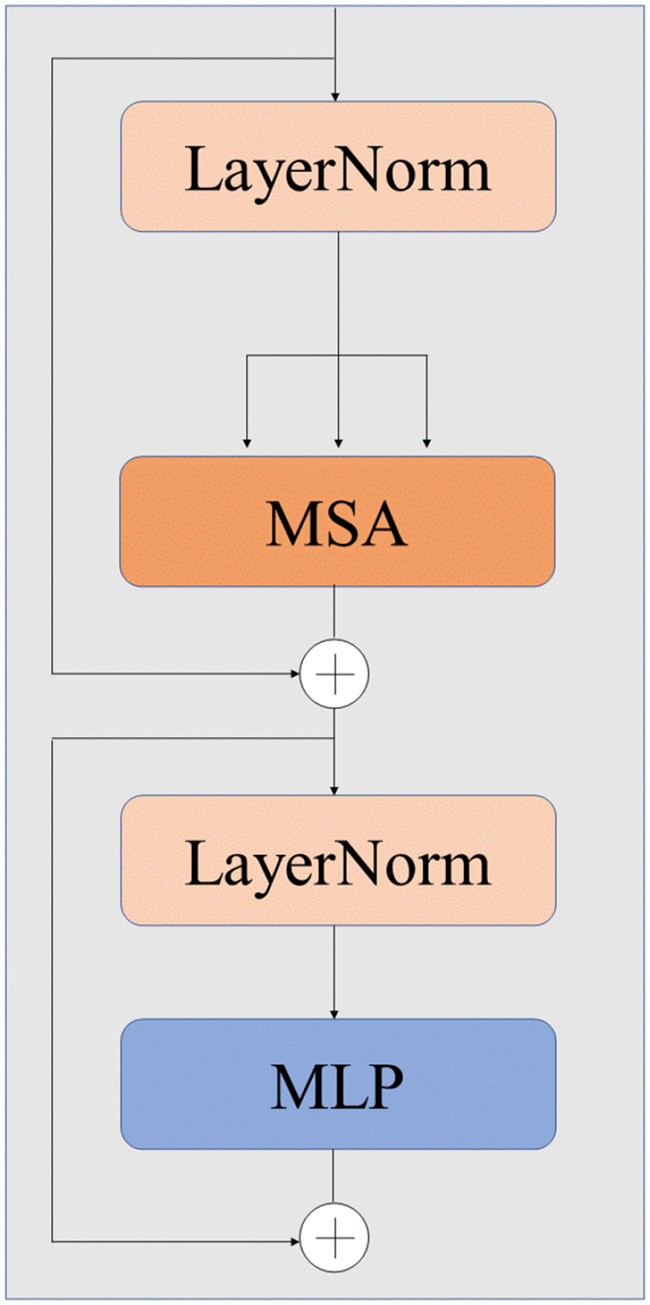
Vision Transformer (ViT) module.

Most of CNN-based medical image fusion model focus on extracting local features of source images. However, the long-range dependence of the source images has not been fully considered, which may result in losing some crucial information from the source images. Compared with CNN, ViT^21^ can better capture global context and long-range dependencies, which aids in modeling more complex visual features. Therefore, ViT is introduced into the proposed Transformer branch in the proposed method to extract the global context multiscale semantic information features from the source images and compensate for the CNN module's inability to capture global information. The ViT module is shown in Figure 3, consists of a patch embedding module, a multihead self-attention layer (MSA) and a multilayer perceptron (MLP).

The core of MSA is the multihead attention mechanism and the scaled-dot product attention module, which are mainly composed of query(*Q*), key(*K*), and value(*V*). The MLP contains two linear layers, each featuring Gelu activation. To introduce the ViT into vision tasks, the source images $X\in R^{C\times H\times W}$ are initially split into a series of several patches $X_{p}=R^{\;p^{2}\times C\times N}$ by ViT. *C*, *H*, and *W* are the channel number, height, and width of the source images, separately. *P* is the width and height of the image patches, *N* represents the total count of image patches, and $N=(H\times W)/P^{2}$ . Then, a linear mapping is used to embed the divided image patches. To acquire the necessary position information, position coding is added to the mapped image patches. The formula for the whole process is:

(1) $$Z_{l}^{\prime }=MSA(LN(Z_{l-1}))+Z_{l-1}\;\;\;\;\;\;\;\;\;\;l=1,\ldots ,L$$

(2) $$Z_{l}=MLP(LN(Z_{l}))+Z_{l}\;\;\;\;\;\;\;\;\;\;l=1,\ldots ,L$$

where $Z_{l}(l=1\ldots L)$ represents the output representation of each Transformer module. *L* represents the total number of Transformer modules. In this paper, *L* is set to 12. By using ViT modules to capture comprehensive information from the original images, including the overall pixel information and spatial structure, the fused result retains a closer overall structure to the source images.

#### Coordinate attention

**Figure 4.**
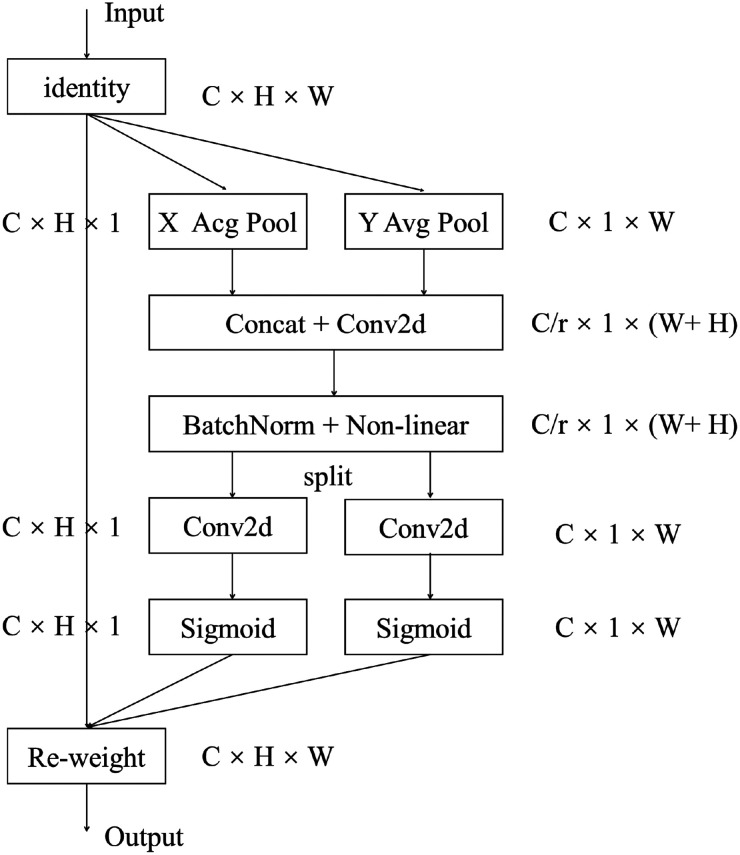
Coordinate attention block.

Ordinary algorithms treat the feature maps of different channels equally, but in fact, the feature information in different channels is different. To improve the focus of the network on the channels that contain important information, the CA^22^ is introduced into the proposed method, as shown in Figure 4. Coordinate attention can capture cross-channel information as well as location-aware and direction-aware details. Accurate location information encodes the channel relationship and long-range dependency, with the process divided into two steps: embedding coordinate information and generating CA. Coordinate information embedding: The global pooling is typically employed to encode spatial information across channels globally, but by compressing global spatial information into channel descriptors, it becomes challenging to preserve location details. To allow the attention module to capture distant spatial interactions with accurate position information, the two-dimensional features are transformed into two parallel one-dimensional features for encoding operation.

#### Subpixel context enhancement module

**Figure 5.**
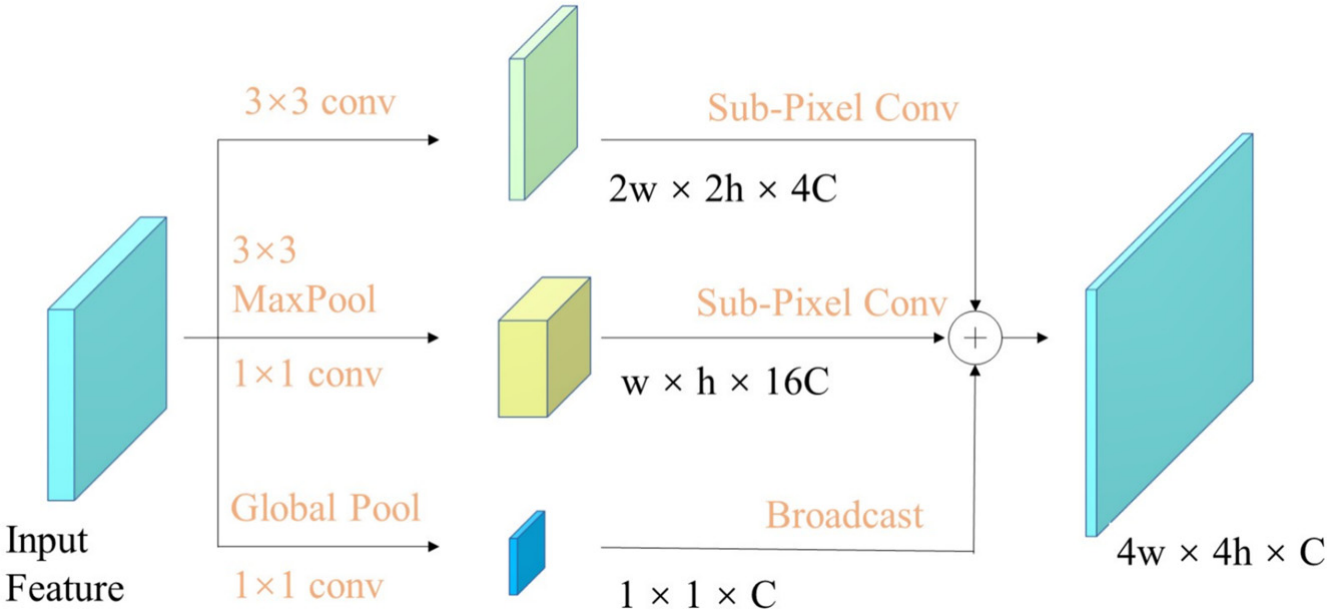
Subpixel context enhancement module.

To extract the source images’ multiscale information, the SCE module^23^ is integrated into the proposed network. The SCE module employs convolution kernels of various sizes to capture multiscale representation adapting to the human visual system's perception. Additionally, the SCE can effectively aggregate and enhance the extracted features, and better integrate local and global information. The structure of SCE is shown in Figure 5. Specifically, the first channel uses 3 × 3 convolution to extract local information. Simultaneously, the channel dimension is transformed to achieve subpixel upsampling. Thereafter, the subpixel convolution is used for two-scale upsampling. The second channel downsampled the input features to *w*/2×*h*/2 through 3 × 3 maximum pooling, and expanded the channel dimension through 1 × 1 convolution. This channel captures abundant contextual information by utilizing a larger receptive field. The third channel performs global average pooling to obtain global context information. Finally, the three generated feature maps are accumulated by mapping and adding them together to obtain an enhanced representation of contextual information, which improves the semantic expression capability of the features.

### Decoder

The decoder performs dimension reduction on the fused features obtained from the feature fusion layer, generating a medical fusion image with lower dimension and less loss of details. The proposed decoder consists of a 3 × 3 convolution kernel, a 1 × 1 convolution kernel, and a *Tanh* activation function. We do not use other layers to avoid the complexity of the training phase and ensure the accurate recovery of the fused image.

### Loss function

A single loss function is limited in optimizing the performance of the proposed fusion model. The hybrid loss can effectively increase the network's convergence rate and improve the fusion performance. The proposed loss can be divided into three parts:

(3) $$L_{\mathrm{total}}=L_{ssim}+\lambda L_{mse}+\lambda L_{tv}$$

Among them, $L_{\mathrm{total}}$ represents the overall loss function. $L_{ssim}$ , $L_{mse}$ , and $L_{tv}$ represent the structural similarity loss, the mean square error loss, and the total variation loss, respectively.

The loss of structural similarity is used to constrain the similarity between the original images and the fused image. The smaller the structural similarity index measure (*SSIM*) loss is, the larger the structural similarity between the input image and fused image. The richer the details, the less information is lost. It is expressed as:

(4) $$L_{ssim}=1-SSIM(I_{\mathrm{out}}-I_{\mathrm{in}})$$

where *I*_out_ is the fused image with the presented network, *I*_in_ is the source medical images. By minimizing $L_{ssim}$ , the model ensures that the fused image retains critical structural details, such as edges and textures, which are essential for maintaining perceptual similarity. This component directly contributes to the structural quality of the fused output.

*Lms*e is used to ensure the content structure of the fused image remains consistent with that of the original medical images. It is defined as:

(5) $$L_{mse}=\frac{1}{M}\overset{i=1}{\underset{M}{\sum }}\;F_{i}-I_{i2}^{2}$$

where *I* represents the source medical image, *F* is the fused image. *i* is the number of fused images. By minimizing the pixel-wise differences, $L_{mse}$ contributes to maintaining the feature fidelity of the fused output. To further eliminate noise, we introduce $L_{tv}$ total change loss^24^ shown following:

(6) $$R(i,j)=I_{\mathrm{out}}(i,j)-I_{\mathrm{in}}(i,j)$$

(7) $$\begin{array}{rl} L_{TV}= & \underset{i,j}{\sum }\;(\|R(i,j+1)-R(i,j){\|}_{2}\\ & \;+\|R(i+1,j)-R(i,j){\|}_{2}) \end{array}$$

where R(i,j) shows the difference between the source medical images and the fused image, $\|\cdot {\|}^{2}$ represents L2−norm , *i* and *j* are, respectively, the horizontal coordinates and vertical coordinates of image pixels. $L_{\mathrm{tv}}$ promotes smoothness while preserving important gradient information, ensuring that the fused image is visually coherent and free from unwanted distortions.

The hybrid loss function employs a weighted multicomponent design to balance structural fidelity, pixel-level accuracy, and gradient smoothness in multimodal medical image fusion. Specifically, the structural similarity loss ( $L_{ssim}$ ) emphasizes edge and texture consistency between the fused image and the source modalities, ensuring the preservation of anatomical details. The mean squared error ( $L_{mse}$ ) penalizes pixel-level discrepancies, reinforcing feature alignment across modalities. The total variation loss ( $L_{\mathrm{tv}}$ ) suppresses high-frequency noise and artifacts, promoting spatial smoothness while retaining important gradient transitions.

In this framework, the hyperparameter λ=20 is used to weight the contributions of $L_{mse}$ and $L_{\mathrm{tv}}$ relative to $L_{ssim}$ . This configuration prioritizes structural similarity through $L_{ssim}$ , while still enforcing content consistency and visual coherence via $L_{mse}$ and $L_{\mathrm{tv}}$ , respectively. The relatively large value of λ amplifies the roles of $L_{mse}$ and $L_{\mathrm{tv}}$ , which is crucial for medical image fusion where both diagnostic feature preservation and artifact suppression are essential. Overall, this hybrid loss effectively guides the network to produce fused images with high perceptual quality, structural consistency, and low noise, thereby improving convergence and fusion performance.

## Experiments

### Experimental configuration and evaluation

In clinical practice, PET-CT has already been integrated into a single imaging modality, providing inherently registered data. Based on a review of the existing literature, PET-MRI, CT-MRI, and SPECT-MRI were selected as dataset. The data set is downloaded from the Harvard whole-brain atlas (http://www.med.harvard.edu), which is widely used in the area of medical image fusion.^25–27^ The dataset consists of 184 pairs of MRI and CT images, 196 pairs of MRI and PET images, and 183 pairs of MRI and SPECT images. The resolution of the images is 224*224, and all image pairs are registered. The downloaded dataset was randomly separated into test and training sets. Specifically, 15 pairs of PET-MRI, CT-MRI, and SPECT-MRI were selected as test sets, and the others as training sets. In addition, the data augmentation (rotation, flip) strategy was used to expand the training data to obtain enough training data and reduce the risk of network overfitting. Specifically, 1521 pairs of CT-MRI images, 1629 pairs of PET-MRI images, and 1512 pairs of SPECT-MRI images were obtained. An NVIDIA GeForce RTX 1080Ti GPU and 16GB of RAM are used to train the proposed fusion network. The batch size, epoch, and learning rate are set as 4, 200, and 1e-4, separately. To speed up the convergence of the network, Adam^28^ optimizer was used.

To assess the proposed model quantitatively, six widely used metrics are selected. They are the *SSIM*,^29^ the multiscale *SSIM* (*MS SSIM*),^30^ the standard deviation (*SD*),^31^ entropy (*EN*),^32^ the spatial correlation deviation (*SCD*)^33^ and the correlation coefficient (*CC*).^34^ Structural similarity index measure evaluates the structural similarity between the fused image and the source images based on brightness, similarity, and contrast distortions. A higher *SSIM* indicates less structural loss and distortion. *MS SSIM* evaluates the ability of the fused images to retain structure at multiple resolution levels, which is more in line with the multiscale perceptual properties of the human eye. A higher *MS SSIM* value indicates better retention of structural details and perceptual information. Correlation coefficient measures the linear correlation between the fused image and the reference image at pixel intensity, reflecting the fidelity of global information transfer. A higher *CC* value indicates greater similarity between the fusion image and the source images, reflecting better fusion performance. Entropy mainly assesses the amount of information contained in the fused image. A larger *EN* indicates that the fused image contains more information. Spatial correlation deviation calculates the sum of the differences in *CC*s between the source image and the fused image within a local window, and assesses the ability of the fusion result to integrate multimodal information. A higher *SCD* indicates better image fusion quality.

### Experiment results and analysis

To emphasize the benefits of the proposed TVNet, TVNet is compared with seven state-of-the-art methods: MSDNet^18^: A network that extracts multiscale features using convolution kernels of different sizes; PMGI^19^: A network that achieves proportionally preserving feature extraction through gradient paths and intensity paths; U2fusion^35^: A unified unsupervised image fusion network; SDNet^18^: A network that proposes a compression-decomposition network and an adaptive decision module based on PMGI to further enhance the fusion performance; EMFusion^36^: An enhanced network that boosts information retention by imposing both surface and deep constraints; MSDRA^37^: A multiscale double-branch residual attention network for anatomical-functional medical image fusion; SwinFuse^38^: A fusion network based on residual Swin Transformer blocks. These methods are trained and tested using the same data set as the proposed TVNet, and the parameters remain the default values used by the original authors.

#### Computed tomography-Magnetic resonance imaging

**Figure 6.**
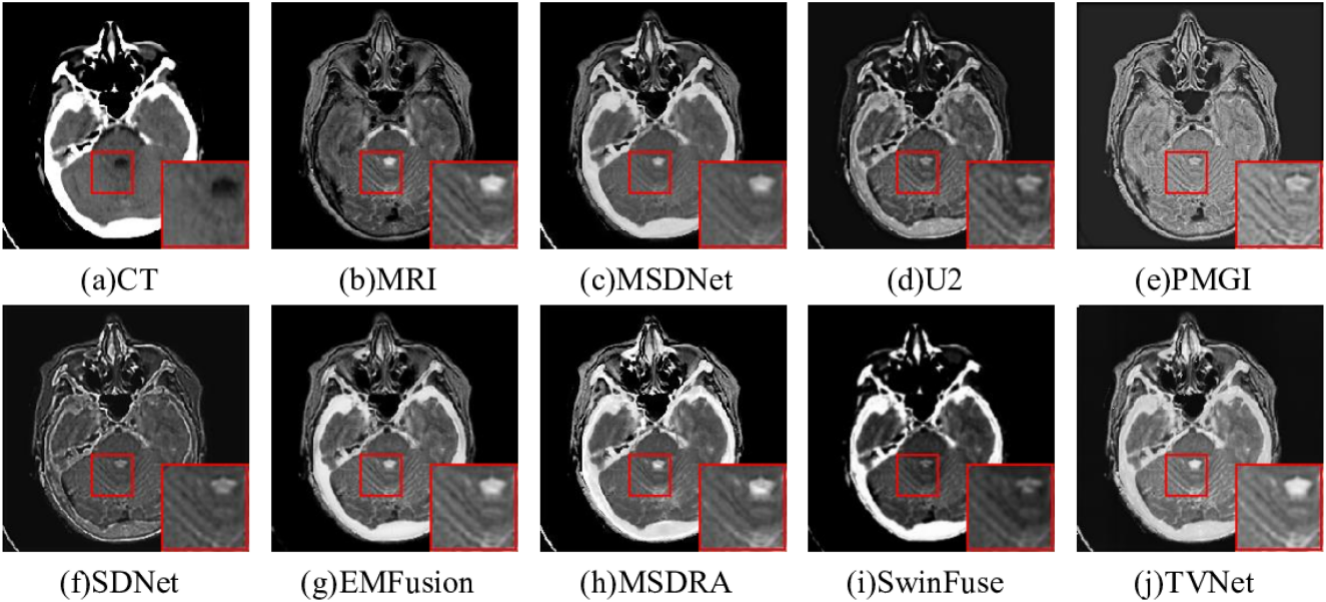
Comparison on computed tomography-magnetic resonance imaging (CT-MRI) fusion.

The fusion results of CT-MRI using various methods are demonstrated in Figure 6. The background of Figure 6(e) is gray black, the contrast is lower, and the dense structure in CT is lost. Compared to other methods, the brightness of Figure 6(c), (d), and (f) is too low, resulting in unclear image details. In addition, the detail information of Figure 6(i) almost disappears, and the texture details in the source MRI are almost lost. The detailed information of Figure 6(g) and (h) is better than other results, but Figure 6(j) retain more of the soft tissue structure in the source images and reflect the abnormal gray values of the lesion area. Table 1 shows the value of the six metrics with different models. As shown in Table 1, the best metrics are highlighted in bold. This notation is consistently applied in the remaining tables. For CT-MRI image pairs, the proposed TVNet obtained the optimal results on *SSIM*, *EN*, *SCD*, *CC,* and *SD* metrics, which indicated that our results achieved the maximum similarity to the source images with less distortion. Although *MS SSIM* metric is not optimal, it is superior to most other methods and is a suboptimal result. This is because our fusion results retained more texture details in MRI, leading to a lower with CT image similarity. However, the fused images generated by the proposed method demonstrate superior on the *SSIM* metric, indicating that our proposed method retains more valuable texture details.

**Table 1.** Comparison on CT-MRI fusion.

Method	MS SSIM	SSIM	EN	SCD	CC	SD
MSDnet	0.9029	0.9065	4.7135	1.2009	0.7991	10.0664
U2fusion	0.8794	0.9174	4.8150	0.7508	0.8150	9.4947
PMGI	0.5595	0.7631	5.2341	0.5418	0.7365	9.7287
SDNet	0.8632	0.9077	4.9160	0.0401	0.6006	9.6999
EMFusion	0.9019	0.9111	4.6567	1.3574	0.8115	9.9876
MSDRA	**0**.**9143**	0.8847	4.4434	1.3644	0.7893	10.0074
SwinFuse	0.9076	0.8554	3.9410	0.8307	0.7293	8.3426
Proposed	0.9120	**0**.**9208**	**5**.**5208**	**1**.**5072**	**0**.**8286**	**10**.**1565**

CC: correlation coefficient; CT-MRI: computed tomography-magnetic resonance imaging; EN: entropy; SCD: spatial correlation deviation; SD: standard deviation; SSIM: structural similarity index measure. Bold indicates the optimal value.

#### positron emission tomography-Magnetic resonance imaging

**Figure 7.**
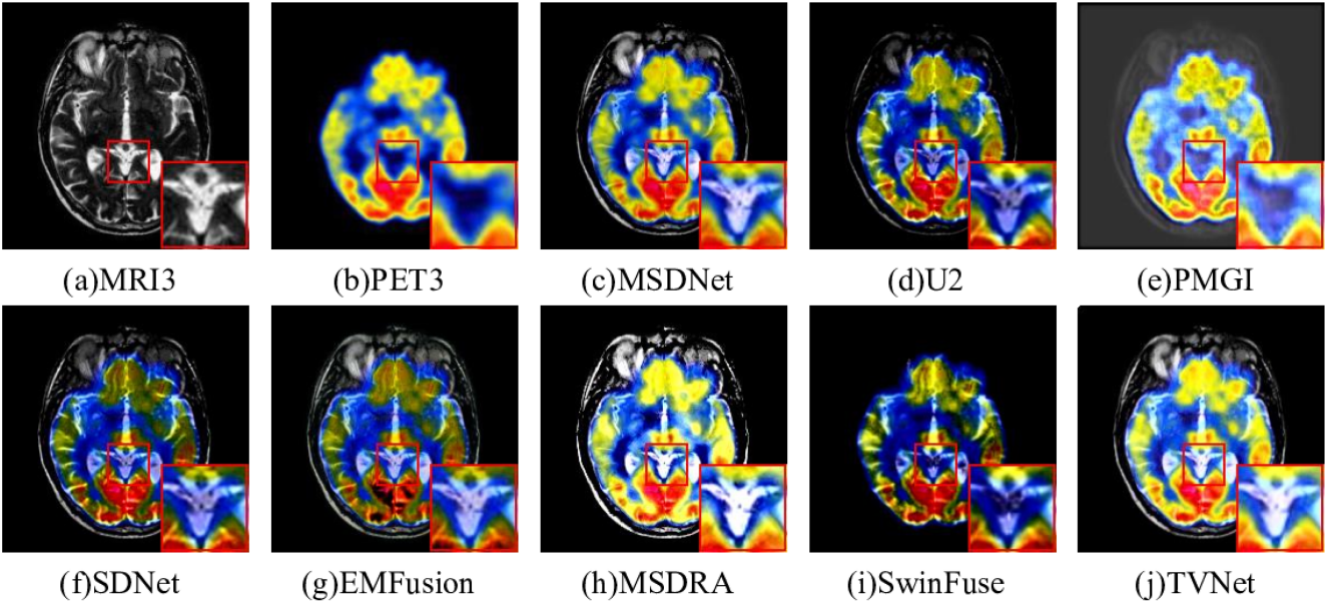
Comparison on magnetic resonance imaging (PET-MRI) fusion.

The fusion results of PET-MRI by different models are illustrated in Figure 7. Figure 7(e) and (i) lose a lot of structure information, and Figure 7(e) is unable to fuse the central sulcus in the MRI brain. Figure 7(c) has a bit of interference in the brain gullies. Figure 7(d) has large noise in the fused image. Figure 7(f) and (g) lose the color information in PET, such as the golden yellow area in the enlarged area. The color fidelity of Figure 7(h) is better than that of other algorithms. However, through careful observation, it was found that the overall brightness of Figure 7(h) is too high to cause the loss of some anatomical details in the MRI. Compared with Figure 7(h), Figure 7(j) not only preserves the functional metabolism and color information in the original PET image but also retains the brightness information of the brain area.

The quantitative evaluation of fused PET-MRI images by different methods is shown in Table 2. In addition to structural similarity *SSIM*, it is the best result in the other five metrics (*MS SSIM, EN, SCD, CC*, and *SD*). These results show that the proposed method can retain the anatomical information in the MRI and the functional information in the PET by preserving a high degree of similarity with the source images.

**Table 2.** Comparison on PET-MRI fusion.

Method	MS SSIM	SSIM	EN	SCD	CC	SD
MSDNet	0.9169	0.9260	4.3017	1.3400	0.8217	8.6551
U2fusion	0.9225	0.9190	3.6209	1.1940	0.8234	7.9213
PMGI	0.7028	0.6716	4.8858	0.6694	0.7328	8.3310
SDNet	0.8848	0.9332	3.8005	0.9201	0.8058	8.1601
EMFusion	0.8875	**0**.**9433**	4.2070	1.1659	0.8041	8.4329
MSDRA	0.8650	0.8484	3.6282	1.5036	0.7935	8.6085
SwinFuse	0.8198	0.8299	3.3600	0.6831	0.7815	7.3353
Proposed	**0**.**9462**	0.9366	**5**.**1585**	**1**.**7938**	**0**.**8399**	**8**.**7592**

CC: correlation coefficient; EN: entropy; PET-MRI: positron emission tomography-magnetic resonance imaging; SCD: spatial correlation deviation; SD: standard deviation; SSIM: structural similarity index measure. Bold indicates the optimal value.

#### Single-photon emission computed tomography-Magnetic resonance imaging

**Figure 8.**
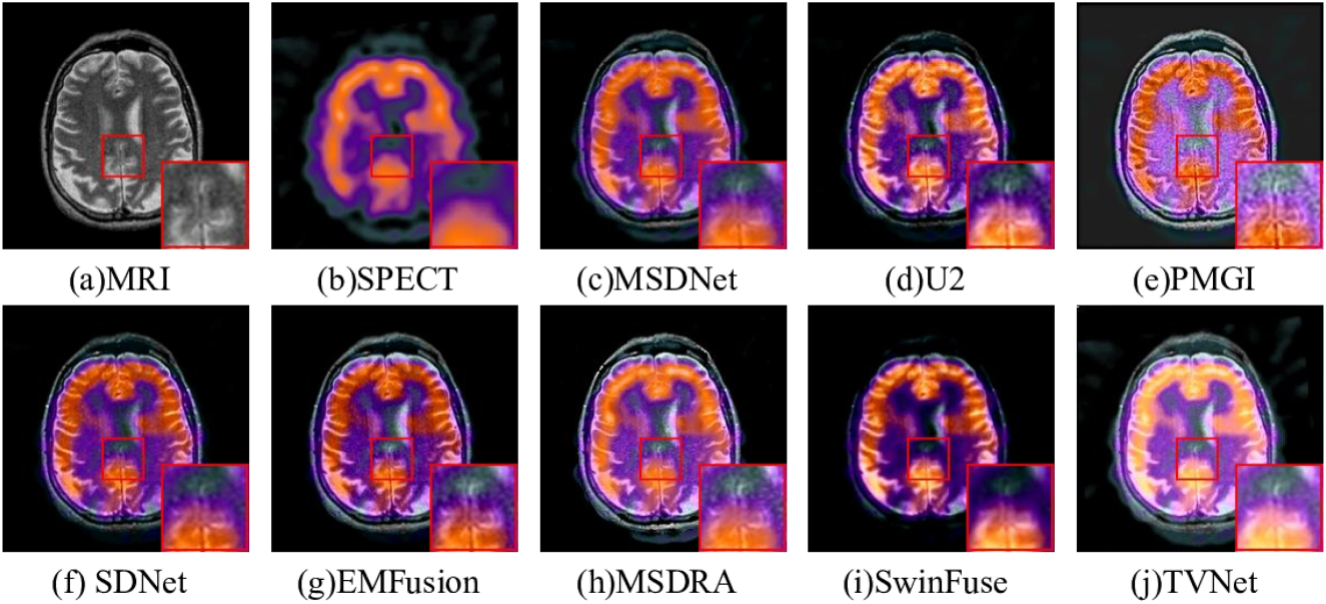
Comparison on single-photon emission computed tomography-magnetic resonance imaging (SPECT-MRI) fusion.

The fusion results of SPECT-MRI by different models are illustrated in Figure 8. The fine white stripes are not obvious in the middle of Figure 8(c) and (f). Although Figure 8(e), (d), and (h) can extract structural information in MRI well, they introduce undesirable noise, for example, white matter blur. Figure 8(i) focuses too much on the color information in SPECT and ignores the soft tissue information in MRI. Figure 8(g) shows that the EMFusion method has good soft tissue maintenance ability, but the color is slightly distorted. Figure 8(j) retains the MRI image details in the fusion result without being overshadowed by the color data from the SPECT image, ensuring better preservation of edge and detail information. As shown in Table 3, the proposed TVNet outperforms other approaches, achieving the best results across all evaluation metrics.

**Table 3.** Comparison on SPECT-MRI fusion.

Method	MS SSIM	SSIM	EN	SCD	CC	SD
MSDNet	0.9317	0.9288	5.4544	0.8950	0.9055	9.6946
U2fusion	0.9180	0.9165	4.7768	1.3322	0.8960	8.9360
PMGI	0.8192	0.8463	5.7294	0.7111	0.8292	9.7428
SDNet	0.9257	0.9289	4.7627	0.7117	0.8894	9.4832
EMFusion	0.9174	0.9311	5.0459	0.6434	0.8630	9.7034
MSDRA	0.8985	0.8683	4.4765	1.3306	0.8681	9.7367
SwinFuse	0.8365	0.8424	4.3899	0.5945	0.8679	8.4128
Proposed	**0**.**9363**	**0**.**9346**	**6**.**2803**	**1**.**8146**	**0**.**9167**	**9**.**9408**

CC: correlation coefficient; EN: entropy; SCD: spatial correlation deviation; SD: standard deviation; SPECT-MRI: single-photon emission computed tomography-magnetic resonance imaging; SSIM: structural similarity index measure. Bold indicates the optimal value.

#### Fusion performance of different disease images

**Figure 9.**
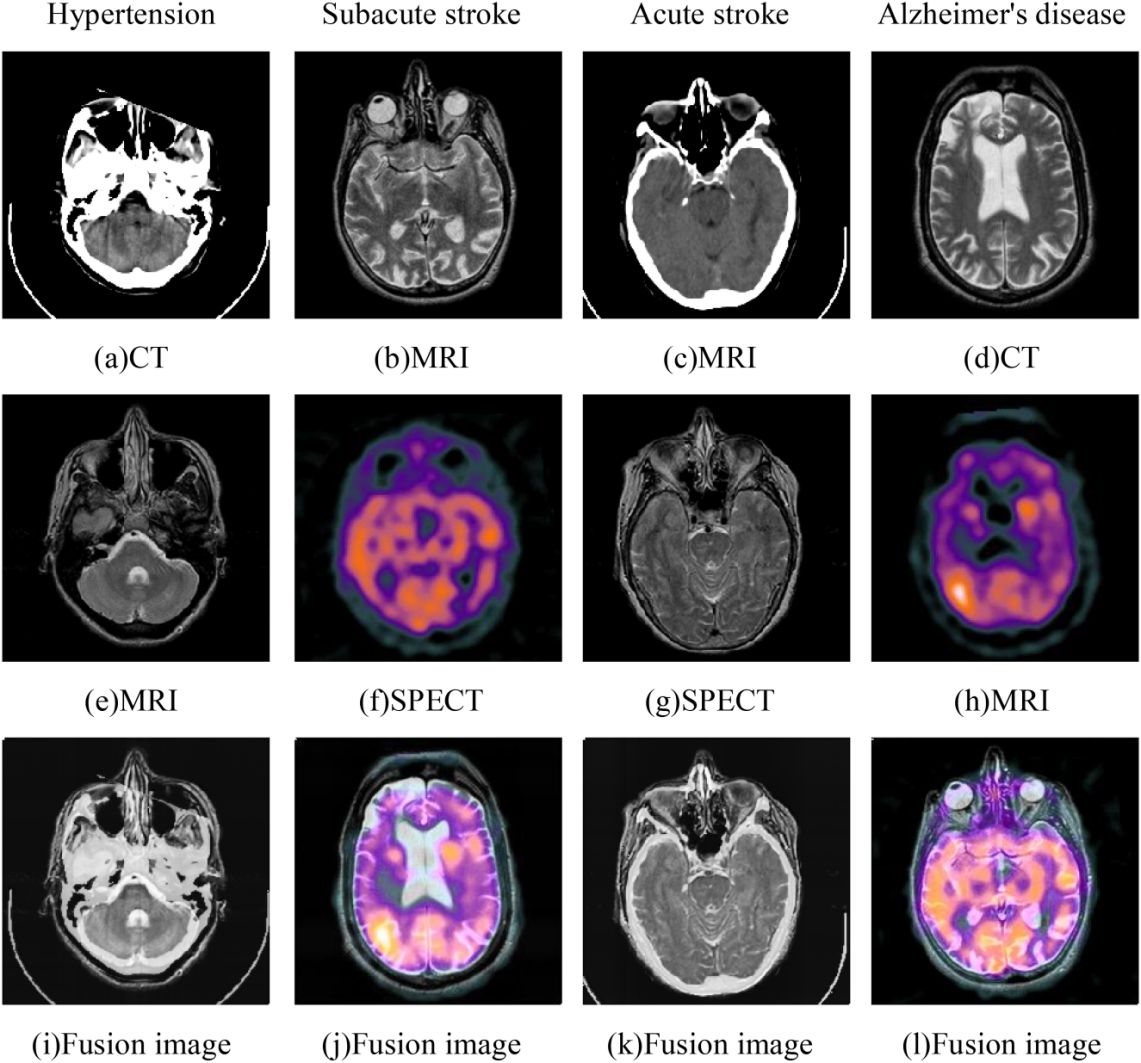
Fusion performance of different disease images.

In order to demonstrate the performance of the proposed fusion network more intuitively, we take the fusion images of brain diseases (hypertension, subacute stroke, acute stroke, and Alzheimer's disease) as an example for analysis shown in Figure 9. In hypertension cases, the fusion results not only preserve the fine structure of cerebral blood vessels but also clearly highlight the boundaries of the basal ganglia. In subacute cases, the proposed method maintains the anatomical details of the ventricular system. In acute stroke cases, the fused images not only preserve the soft tissue edge details of the original MRI scans but also enhance the salient features of specific lesion regions. The fusion results of Alzheimer's disease cases successfully capture the subtle atrophy features of the hippocampus. In general, the proposed method can effectively integrate the key features of multimodal medical images and significantly enhance the contrast of the lesion area while maintaining the clarity of anatomical structures.

### Ablation experiment

#### Ablation analysis for two branches

**Figure 10.**
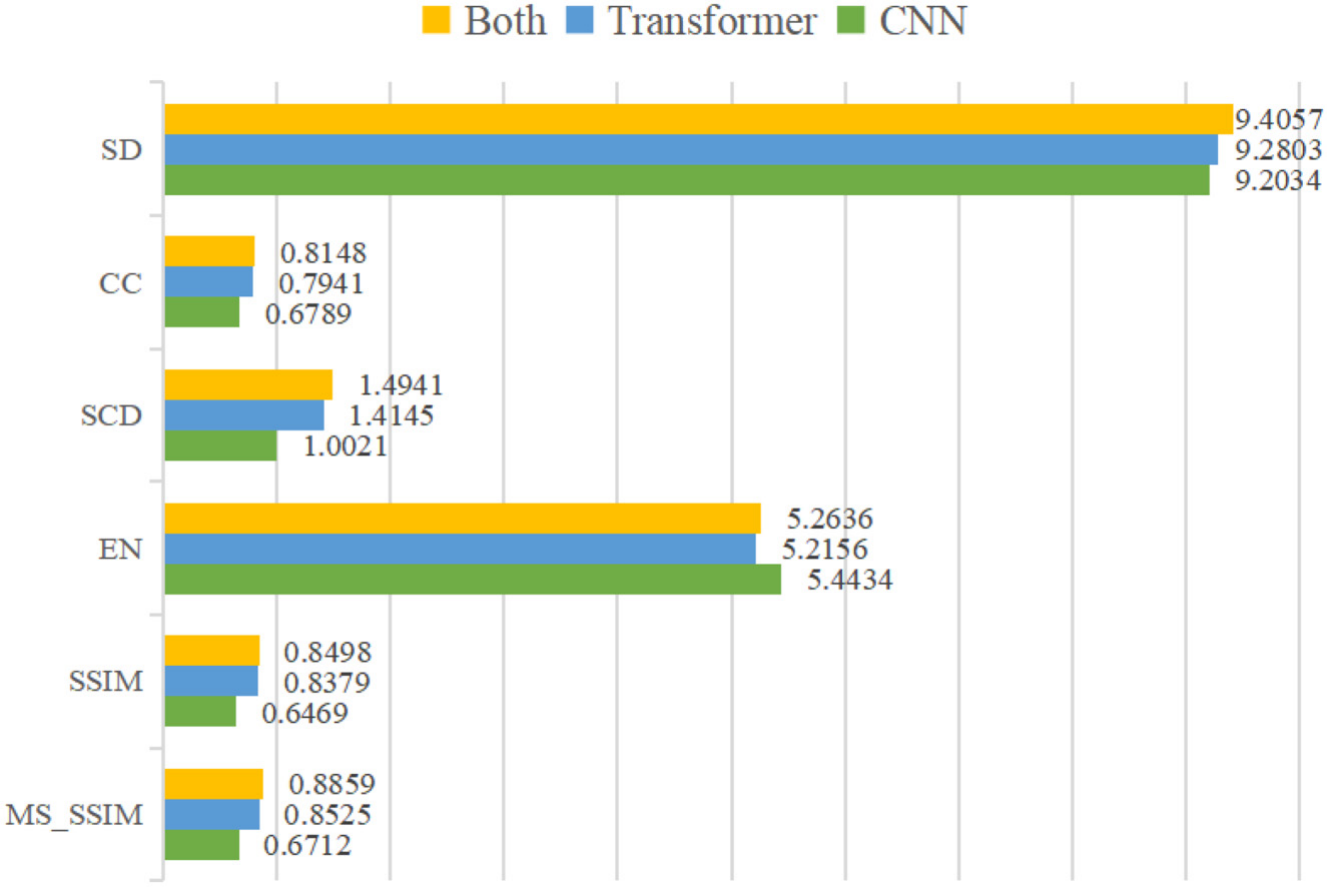
Histogram of average values of six metrics.

To assess the effectiveness of feature extraction in the two branches of the encoder, three experiments are conducted: 1) Single CNN branch fusion model: The encoder only uses CNN branch, specifically, only OSA module is used to extract features. 2) Single Transformer branch fusion model: The encoder only uses Transformer branch, specifically, only ViT module is used to extract features. 3) The proposed dual-branch fusion model. Figure 10 shows the histogram of average values of five metrics derived in 45 pairs of multimodal medical images (PET-MRI, CT-MRI, and SPECT-MRI). It is evident that the fused images obtained by dual-branch model combining CNN branch, and Transformer branch for feature extractor is better than the fused image by the other two models only using CNN branch or Transformer branch to the extract feature.

In terms of the *MS SSIM* metric, the dual-branch model achieves a score of 0.8859, showing a 3.9% improvement over the Transformer (0.8525) and a 32.0% increase compared to the CNN (0.6712). For the *SSIM* metric, the dual-branch model scores 0.8498, exceeding the Transformer (0.8379) by 1.4% and the CNN (0.6469) by 31.4%. When using only the CNN branch, both *SSIM* and *MS SSIM* scores are relatively low, primarily due to the convolutional structure's limited ability to model long-range dependencies and capture global structural information, resulting in insufficient preservation of structural details from the source images. Although the Transformer branch performs better than CNN in terms of structural similarity thanks to its global modeling capacity, it may overlook fine-grained texture and edge information due to the lack of precise local feature extraction. The dual-branch architecture combines the global perception capability of the Transformer with the strong local detail extraction of the CNN, effectively compensating for their individual shortcomings. This synergy leads to improved structural similarity and perceptual quality of the fused images.

The dual-branch model achieves the highest performance on the *SD* metric, with a score of 1.4941, surpassing the Transformer (1.4145) by 5.6% and the CNN (1.0021) by 49.1%. Similarly, it achieves a *CC* score of 0.8148, which is 2.6% higher than the Transformer (0.7941) and 20.0% higher than the CNN (0.6789). It can be concluded that using the dual-branch combining CNN branch with Transformer branch as feature extractor can fully integrate the texture details extracted by OSA block and structure information extracted by ViT block to achieve better fusion effect.

#### Ablation study for different modules

**Figure 11.**
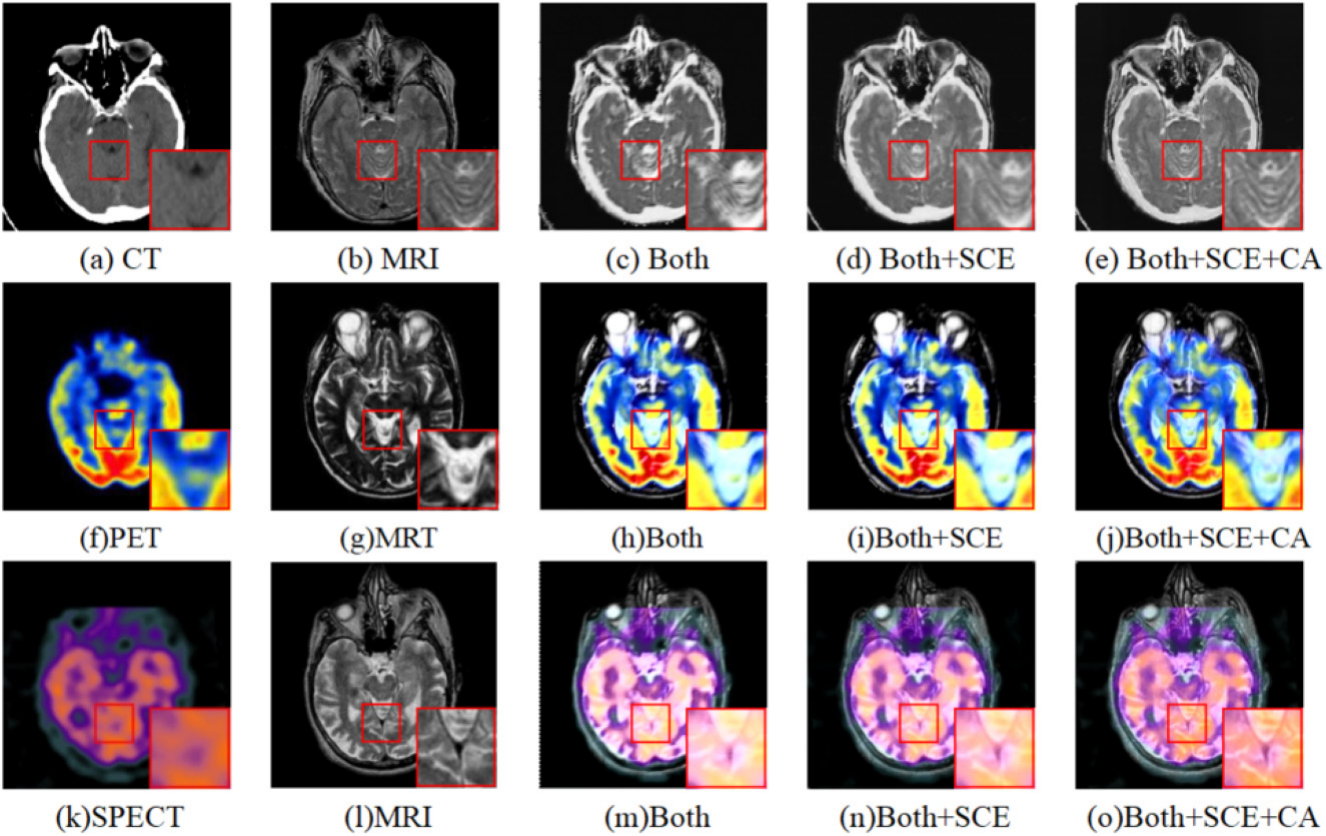
Fused images of computed tomography-magnetic resonance imaging (CT-MRI), positron emission tomography-magnetic resonance imaging (PET-MRI), and single-photon emission computed tomography-magnetic resonance imaging (SPECT-MRI) using different modules.

To demonstrate the effectiveness of SCE module and CA module, they are progressively added on basis of the dual-branch model. Tables 4, 5, and 6 show the quantitative evaluation results based on different modules on CT-MRI, PET-MRI, and SPECT-MRI. The best metrics are bold. Figure 11 shows the fusion results by adding different modules. The first and second columns are source images, the third column “Both” indicates the result of dual-branch fusion model, and the fourth column “Both + SCE” indicates that the SCE enhancement module is added on the dual-branch model. The fifth column “Both + SCE + CA” indicates that CA module is added on the basis of “Both + SCE.”

**Table 4.** CT-MRI fusion results using different modules.

Method	MS SSIM	SSIM	EN	SCD	CC	SD
Both	0.8703	0.8389	5.1138	1.2509	0.7614	10.1080
Both + SCE	**0**.**9144**	0.9119	5.3703	**1**.**5766**	0.8257	10.1209
Both + SCE + CA	0.9120	**0**.**9208**	**5**.**5208**	1.5072	**0**.**8286**	**10**.**1565**

CA: coordinate attention; CC: correlation coefficient; CT-MRI: computed tomography-magnetic resonance imaging; EN: entropy; SCD: spatial correlation deviation; SCE: Sub-pixel Context Enhancement; SD: standard deviation; SSIM: structural similarity index measure. Bold indicates the optimal value.

**Table 5.** PET-MRI fusion results using different modules.

Method	MS SSIM	SSIM	EN	SCD	CC	SD
Both	0.9020	0.8609	4.7545	1.5910	0.7970	8.5180
Both + SCE	0.9454	0.9221	4.9642	1.7116	0.8258	8.6592
Both + SCE + CA	**0**.**9462**	**0**.**9366**	**5**.**1585**	**1**.**7938**	**0**.**8399**	**8**.**7592**

CA: coordinate attention; CC: correlation coefficient; EN: entropy; PET-MRI: positron emission tomography-magnetic resonance imaging; SCD: spatial correlation deviation; SCE: Sub-pixel Context Enhancement; SD: standard deviation; SSIM: structural similarity index measure. Bold indicates the optimal value.

**Table 6.** SPECT-MRI fusion results using different modules.

Method	MS SSIM	SSIM	EN	SCD	CC	SD
Both	0.8853	0.8497	5.9226	1.6403	0.8860	9.5911
Both + SCE	0.9246	0.9103	6.2287	1.7628	0.9069	9.5884
Both + SCE + CA	**0**.**9363**	**0**.**9346**	**6**.**2803**	**1**.**8146**	**0**.**9167**	**9**.**9408**

CA: coordinate attention; CC: correlation coefficient; EN: entropy; SCD: spatial correlation deviation; SCE: Sub-pixel Context Enhancement; SD: standard deviation; SPECT-MRI: single-photon emission computed tomography-magnetic resonance imaging; SSIM: structural similarity index measure. Bold indicates the optimal value.

For CT-MRI fusion, resulting from the interference of the original CT image, Figure 11(c) lost the information of the lesion regions presented in the original MRI, resulting in an unclear fused image. Figure 11(d) preserved more detailed information than Figure 11(c), but Figure 11(d) was blurred. Figure 11(e) not only maintained the lesion area information with suitable abnormal gray values from the source CT image but also fully retained the soft tissue structure details from the source MRI. For PET-MRI fusion and SPECT-MRI fusion, Both Figure 11(h) and (m) lost the original MRI structural information. Compared with Figure 11(c) and (h), Figure 11(d) and (n) contained more detailed information, but they also preserve the structural details of the brain grooves. Obviously, the structures and details of Figure 11(j) and (o) are the clearest, which not only contain the color information of PET but also present the anatomical details well.

From Table 4, 5, and 6, it can be observed that in terms of objective evaluation metrics, “Both + SCE” shows significant improvement over “Both” in each metric. It indicates that the SCE enhancement module is effective in aggregating local and global features. The fusion performance is further improved in “Both + SCE + CA,” which indicates that the fusion image obtained by allocating different weights using CA can retain more additional details and brightness information from the source images.

#### Ablation study for loss function

**Figure 12.**
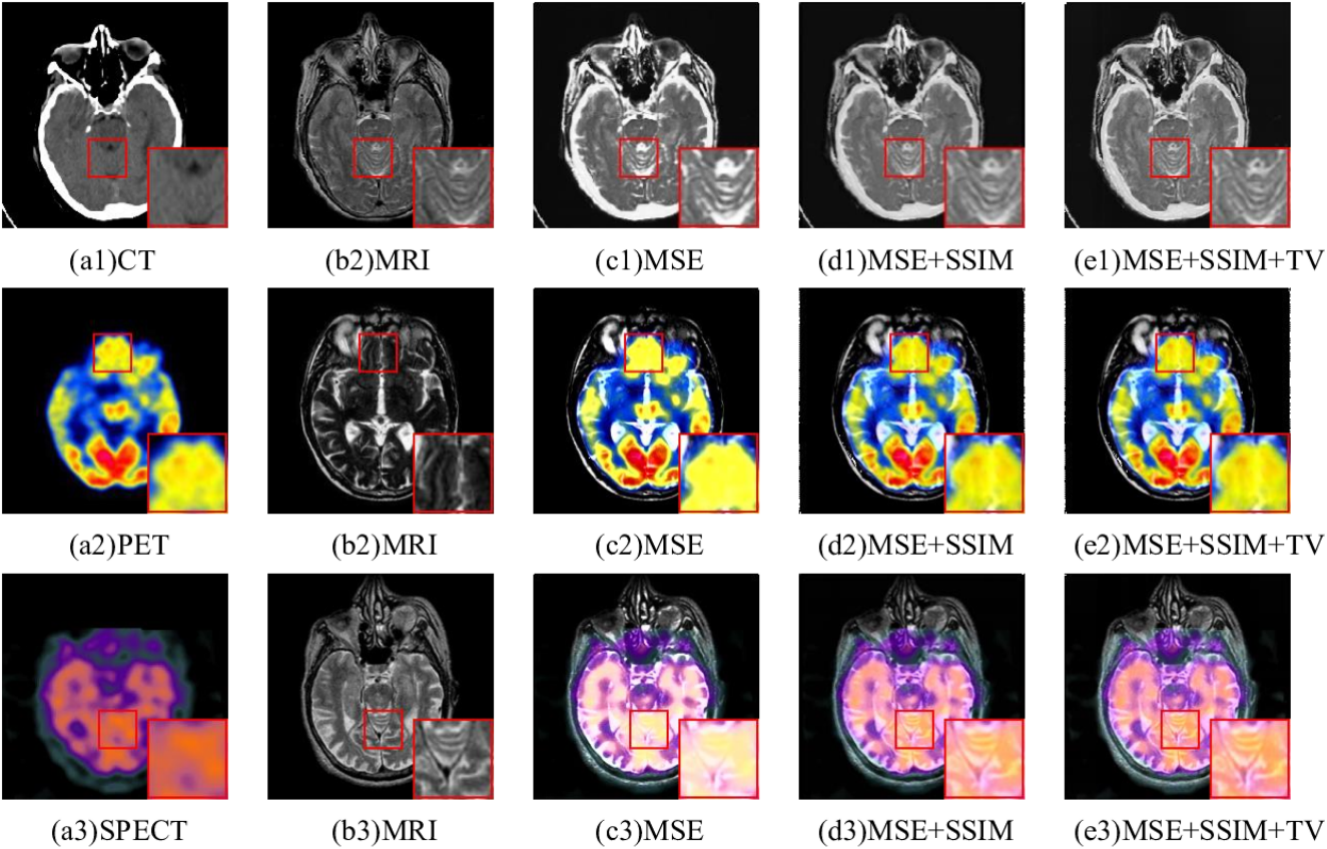
Ablation experiments based on different loss functions.

To demonstrate the efficacy of the proposed loss functions, three different loss functions are verified on the same medical image dataset. The fusion images of CT-MRI by different loss functions are shown in Figure 12. A single MSE loss function made the fusion results overly bright and information about the soft tissue structure in MRI is lost. The edges of the middle focus region of Figure 12(d1) are blurred. For the PET-MRI and SPECT-MRI, the fused image using a single MSE loss function contained the least detail and structure information. Figure 12(d2) and (e3) incorporated more structural details, but the structural edge texture of MRI in the enlarged region was not clear. Clearly, in comparison to other fusion results, Figure 12(e2) and (e3) both preserved the color information from the original images and preserved the texture details from the MRI.

As can be seen from Table 7, 8, and 9, different loss functions can produce different fusion results. In Table 7, 8, and 9, the top metrics are highlighted in bold black text. According to the first two rows in the Table 7, 8, and 9, it can be seen that *SSIM* loss function can improve *MS SSIM*, *SSIM*, *EN*, *CC*, and *SD* metrics of fused images, but reduce *SCD* metric. Figure 12(d1) to (d3) obviously contain more details and texture information than Figure 12(c1) to (c3), which proves that the *SSIM* loss function has the ability to enhance details and edges. From the second and third rows, it can be observed that *TV Loss* improved all metrics. Although the result on *SCD* metric is not the best, it also achieves the second-best result. Figure 12(e1) to (e3) is clearer than Figure 12(c1) to (c3). It is proved that the *TV* loss function has the ability to enhance image details and reduce noise. To sum up, different loss functions exhibit obvious differences regarding both subjective and objective evaluations on fused images. Obviously, the hybrid loss functions introduced can greatly enhance fusion effects, including detail preservation, edge strength, and color fidelity. However, a single loss function is unable to improve all of these aspects at once.

**Table 7.** CT-MRI fusion comparison using different loss functions.

Method	MS SSIM	SSIM	EN	SCD	CC	SD
MSE	0.8980	0.8989	5.1373	**1**.**5215**	0.8065	9.9347
MSE + SSIM	0.9115	0.9138	5.2730	1.4568	0.8247	10.1014
MSE + SSIM + TV	**0**.**9120**	**0**.**9208**	**5**.**5208**	1.5072	**0**.**8286**	**10**.**1565**

CC: correlation coefficient; CT-MRI: computed tomography-magnetic resonance imaging; EN: entropy; SCD: spatial correlation deviation; SD: standard deviation; SSIM: structural similarity index measure. Bold indicates the optimal value.

**Table 8.** PET-MRI fusion comparison using different loss functions.

Method	MS SSIM	SSIM	EN	SCD	CC	SD
MSE	0.9133	0.8937	3.8635	**1**.**8078**	0.8236	8.2875
MSE + SSIM	0.9404	0.9269	4.9068	1.6082	0.8139	8.7374
MSE + SSIM + TV	**0**.**9462**	**0**.**9366**	**5**.**1585**	1.7938	**0**.**8399**	**8**.**7592**

CC: correlation coefficient; EN: entropy; PET-MRI: positron emission tomography-magnetic resonance imaging; SCD: spatial correlation deviation; SD: standard deviation; SSIM: structural similarity index measure. Bold indicates the optimal value.

**Table 9.** SPECT-MRI fusion comparison using different loss functions.

Method	MS SSIM	SSIM	EN	SCD	CC	SD
MSE	0.8971	0.8925	5.5070	**1**.**8465**	0.9060	9.4474
MSE + SSIM	0.9378	0.9219	6.1915	1.6898	0.9091	9.5602
MSE + SSIM + TV	**0**.**9363**	**0**.**9346**	**6**.**2803**	1.8146	**0**.**9167**	**9**.**9408**

CC: correlation coefficient; EN: entropy; SCD: spatial correlation deviation; SD: standard deviation; SPECT-MRI: single-photon emission computed tomography-magnetic resonance imaging; SSIM: structural similarity index measure. Bold indicates the optimal value.

## Discussion

TVNet performs well in multimodal medical image fusion tasks such as CT-MRI, PET-MRI, and SPECT-MRI, which is mainly attributed to its dual-branch architecture. This architecture combines the advantages of the CNN branch based on the OSA module (for capturing fine-grained texture details) and the ViT branch (for modeling long-range dependencies), while the SCE module provides multiresolution perception capabilities, and the CA module enhances the focus on key channels and spatial regions, especially achieving remarkable results in preserving diagnosis-related features such as lesion areas and tissue boundaries. Moreover, the hybrid loss function of multiobjective optimization design forces the alignment of edges and anatomical contours through $L_{ssim}$ to ensure structural consistency. The $L_{mse}$ term reduces pixel-wise intensity discrepancies to preserve modality fidelity, and the $L_{\mathrm{tv}}$ term suppresses noise and enhances smoothness, especially in homogeneous regions. TVNet performs well in multimodal medical image fusion tasks such as CT-MRI, PET-MRI, and SPECT-MRI, which is mainly attributed to its dual-branch architecture design. This architecture combines the advantages of the CNN branch based on the OSA module (for capturing fine-grained texture details) and the ViT branch (for modeling remote dependencies), while the SCE module provides multiresolution perception capabilities, and the CA module enhances the focus on key channels and spatial regions, especially achieving remarkable results in preserving diagnosis-related features such as lesion areas and tissue boundaries.

Interestingly, our proposed method achieves the best overall performance on SPECT-MRI fusion, especially in terms of *EN*, *SCD*, and *CC*. This may be attributed to the complementary nature of MRI's high-resolution anatomical information and SPECT's functional signals, which often exhibit sparse but salient metabolic regions. The dual-branch architecture and residual fusion module are particularly effective in capturing and preserving such complementary features. Although previous fusion studies, such as PMGI and U2Fusion, have reported performance variations across modality pairs, few have explicitly analyzed or explained them. Our results suggest that modality-specific characteristics can significantly affect fusion outcomes, and future research may benefit from designing adaptive mechanisms tailored to different modality combinations.

Despite the promising results, several limitations remain. First, the dual-branch architecture leads to high computational costs, which can hinder deployment in real-world clinical settings. Second, although the proposed method performs well on selected datasets, it has not yet been applied to clinical evaluation or diagnosis across a range of neurological diseases. Future research may focus on developing lightweight network variants and adaptive fusion mechanisms, as well as incorporating disease-specific priors to enhance clinical applicability and generalization.

## Conclusions

In this paper, we proposed TVNet, a novel dual-branch feature enhancement network for multimodal medical image fusion. The model integrates the advantages of OSA modules and ViTs by employing a dual-branch encoder. The CNN branch with OSA modules effectively captures fine-grained local textures and detail information, while the Transformer branch is capable of modeling global dependencies and capturing structural semantics. Furthermore, the introduction of the CA mechanism enables the network to emphasize regions of interest and suppress less informative areas, enhancing the feature representation. The SCE module further enriches the multiscale feature integration, allowing better preservation of both anatomical structure and functional information. A hybrid loss function combining structural, feature-level, and gradient-level components was designed to ensure the structural fidelity and visual clarity of the fused images. Extensive experiments on PET-MRI, CT-MRI, and SPECT-MRI datasets demonstrate that TVNet consistently outperforms seven state-of-the-art fusion methods in terms of both subjective visual quality and objective evaluation metrics such as *SSIM*, *MS SSIM*, *CC*, and *SD*. Particularly, the model showed the most significant improvement on the SPECT-MRI dataset, suggesting its strong ability to fuse highly complementary imaging modalities. For future work, we plan to introduce self-supervised learning approaches for pretraining the encoder branches, enabling the model to learn more generalizable and robust feature representations from unlabeled multimodal medical data.
